# Changes in serum albumin and other nutritional markers when using sucroferric oxyhydroxide as phosphate binder among hemodialysis patients: a historical cohort study

**DOI:** 10.1186/s12882-019-1582-9

**Published:** 2019-10-29

**Authors:** Kamyar Kalantar-Zadeh, Linda H. Ficociello, Vidhya Parameswaran, Nicolaos V. Athienites, Claudy Mullon, Robert J. Kossmann, Daniel W. Coyne

**Affiliations:** 1 Irvine School of Medicine, University of California, Orange, CA USA; 2 Fresenius Medical Care Renal Therapies Group, Waltham, MA USA; 3 Renal Medical Care PC, Brockton, MA USA; 4 Washington University School of Medicine, 660 S. Euclid Ave., CB 8129, St. Louis, MO 63110 USA

**Keywords:** Hemodialysis, Albumin, Sucroferric oxyhydroxide, Phosphorous, Phosphate binder

## Abstract

**Background:**

Elevated serum phosphorus concentrations are common among maintenance hemodialysis patients. Protein is a major source of dietary phosphate, but restriction of protein intake can result in hypoalbuminemia and protein-energy wasting. We hypothesized that sucroferric oxyhydroxide (SO), a potent phosphate binder with a low pill burden, may reduce serum phosphorus levels in hemodialysis patients with hypoalbuminemia without adversely impacting albumin levels or dietary intake of protein.

**Methods:**

We retrospectively examined de-identified data from 79 adult, in-center hemodialysis patients with baseline hypoalbuminemia (≤ 3.5 g/dL) switched to SO as part of routine clinical care for at least 1 year. Temporal changes (3-month intervals from baseline through Q4) in phosphate binder pill burden, serum phosphorous levels, nutritional markers, and equilibrated Kt/V were analyzed. Data from a matched reference group of non-hypoalbuminemic patients (*N* = 79) switched to SO were also examined.

**Results:**

SO therapy was associated with a mean reduction of 45.7 and 45.1% in daily phosphate binder pill burden, and a mean reduction of 0.4 mg/dL and 0.51 mg/dL in serum phosphorus levels for the hypoalbuminemic and non-hypoalbuminemic patients, respectively. Hypoalbuminemic patients demonstrated significant increases in mean serum albumin levels from 3.50 mg/dL at baseline to 3.69, 3.74, 3.70, and 3.69 mg/dL during Q1 through Q4, respectively (*P* < 0.0001), whereas serum albumin levels remained unchanged in the non-hypoalbuminemic group.

**Conclusions:**

Both hypoalbuminemic and non-hypoalbuminemic patients switching to SO exhibited significant reductions in serum phosphorus concentrations and daily phosphate binder pill burden. Among hypoalbuminemic patients, the initiation of SO therapy was also associated with increases in serum albumin, suggesting therapy may have allowed patients to increase their dietary intake of protein.

## Background

More than one-third of chronic kidney disease patients undergoing hemodialysis (HD) have serum phosphorus (sP) levels > 5.5 mg/dL [1]. The interplay among protein intake, sP, and serum albumin (sAlb) levels raises a clinical conundrum when managing patients’ sP. Hyperphosphatemia and hypoalbuminemia are independent risk factors for mortality among dialysis patients, thus clinicians may be wary of correcting one risk factor at the “expense” of the other [2–5]. Reducing dietary protein consumption, while limiting phosphate intake, can result in reduced sAlb, decreased normalized protein catabolic rate (nPCR), and protein-energy wasting (PEW). These PEW parameters have been associated with increased mortality and reduced quality of life among HD patients [4–10]. For example, the reduction of both sP and sAlb has been associated with a 26% increase in mortality (relative to increases in both parameters) [4]. The relationship between sP and sAlb may be further confounded by the gastrointestinal side effects of some phosphate binders (PBs) [11], which may impair appetite and protein intake. Additionally, use of less effective PB therapy may lead to more dietary restrictions in an attempt to control sP, leading to lower dietary protein intake, higher likelihood of PEW, and poor outcomes [12, 13].

Sucroferric oxyhydroxide (SO; VELPHORO® [Fresenius Medical Care Renal Therapies Group, Waltham, MA, USA]) is a potent PB indicated for the control of sP in dialysis patients, with a starting dose of 3 tablets/day. SO is a non-calcium, chewable, iron-based agent with high phosphate-binding capacity and a starting dose of 3 tablets/day (1500 mg daily) [14, 15]. Over the 1-year follow-up period of a 24-week phase 3 trial and associated 28-week extension study, control of sP was achieved with markedly reduced mean SO pill burden when compared to sevelamer (3.3 vs 8.7 tablets per day) [16]. Retrospective analyses of patients switched from another PB to SO have also demonstrated reductions in pill burden of approximately 50% [14, 17, 18]. Given its high potency in binding phosphate [15], SO use may enable a less restrictive protein diet, leading to correction of hypoalbuminemia and PEW. The current analysis investigates temporal changes in sP and nutritional parameters among hypoalbuminemic (hypoAlb) HD patients prescribed SO as part of routine clinical care. We hypothesized that SO would reduce sP while allowing for improvement in albumin concentration secondary to changes in dietary intake of protein and/or changes in gastrointestinal symptoms.

## Methods

### Study design

This retrospective cohort study utilized de-identified data extracted from the Fresenius Kidney Care clinical data warehouse and a renal pharmacy service (FreseniusRx) database. Adult, in-center HD patients prescribed SO monotherapy as part of routine care and continued SO therapy for ≥12 months were included. Treatment periods were defined as baseline (−Q2, −Q1; 3-month periods before SO) and SO therapy (Q1 to Q4; 12 months of SO). The hypoAlb cohort included patients with sAlb ≤3.5 g/dL at baseline (−Q2 or − Q1). All patients were required to have information on age, sex, race, body mass index, and diabetes status. A reference group of non-hypoalbuminemic (NhypoAlb; sAlb > 3.5 g/dL at baseline) patients was selected with individuals matched to the hypoAlb cohort in a 1:1 ratio on age (±5 years), sex, self-reported race, body mass index (±2 kg/m^2^), and diabetes status.

### Clinical variables and statistical analysis

Clinical parameters of interest included PB pill burden, sP, nutritional markers (sAlb, equilibrated nPCR, weight, and serum creatinine), equilibrated Kt/V, intact parathyroid hormone (iPTH) levels, and corrected serum calcium. SAlb and nPCR were each divided by sP to calculate phosphorus-attuned variables, allowing assessment of the impact of lowering sP without restricting dietary protein intake [18]. Laboratory tests repeated within a month were averaged to overcome short-term measurement variability. Changes in quarterly clinical markers before and after SO switch were examined using linear mixed models to account for repeated measurements. Summary statistics (monthly and quarterly) were presented as least-square (LS) means and standard error (SE). *P* values compared estimates across treatment quarters. Given the limited number of hypoAlb patients in the database meeting elgibility requirements, development of an NhypoAlb cohort matched on additional, potentially confounding variables (e.g., baseline PB) was deemed impractical. As such, formal analyses comparing the hypoAlb and NhypoAlb cohorts were not performed.

## Results

Seventy-nine hypoAlb patients switched to SO were identified in the database; most patients were receiving calcium acetate- or sevelamer-based PBs at baseline. Twelve (15%) of these patients had been undergoing dialysis for fewer than 120 days. Clinical parameters at baseline and across SO follow-up among patients with baseline hypoAlb are presented in Table 2. Prior to SO (−Q1), the mean sP concentration was 6.79 mg/dL and the mean sAlb level was 3.50 g/dL. At -Q1, sP concentrations < 4.5 mg/dl and < 5.5 mg/dl were 2.5 and 24.1% of patients, respectively. At all SO therapy follow-up timepoints, patients in the hypoAlb group demonstrated significant reductions from baseline in PB pill burden and sP. At Q4, patients achieving sP concentrations < 4.5 mg/dl and < 5.5 mg/dl were 15.2 and 38.0%, respectively. A mean sAlb increase of 0.18 g/dL was observed during SO treatment. Concurrent with SO therapy, significant increases in pre- and post-dialysis weight were observed for the hypoAlb cohort.

By design, the NhypoAlb reference group had similar age, sex, race, body mass index, and diabetes status distribution as the hypoAlb cohort. Prior to SO (−Q1), sP concentrations < 4.5 mg/dl and < 5.5 mg/dl were 1.3 and 13.9% of patients, respectively. As detailed in Table 1, mean dialysis vintage was longer for the NhypoAlb patients than for the hypoAlb group (45.3 vs 34.7 months). Significant reductions from baseline in PB pill burden and sP were observed at all SO therapy timepoints (Table 3). At Q4, patients achieving sP concentrations < 4.5 mg/dl and < 5.5 mg/dl were 15.2 and 30.4%, respectively. In contrast to hypoAlb patients, sAlb levels remained unchanged (approximately 4.0 g/dL) throughout most of the SO follow-up period (at Q4, a small but statistically significant reduction was observed). Additionally, the NhypoAlb cohort failed to demonstrate significant changes in serum creatinine or body weight (with the exception of a small increase in pre-dialysis weight observed at Q1). Given the differences in dialysis vintage of patients in the 2 study populations, a sensitivity analysis excluding 14 matched pairs where ≥1 patient had dialysis vintage < 120 days was performed. It revealed mean sAlb changes from baseline of + 0.12 g/dL and + 0.02 g/dL for hypoAlb and NhypoAlb patients, respectively.

**Table 1:** Demographic characteristics

Characteristic	hypoAlb patients (*n* = 79)	NhypoAlb patients (*n* = 79)
Age, years	54.9	55.1
Dialysis vintage, months	34.7	45.3
Incident HD patients,^a^ %	15.2%	3.8%
Male, %	53.2%	53.2%
Race, %		
Black	35.4%	35.4%
White	62.0%	62.0%
Other	2.6%	2.6%
Hispanic/Latino, %	11.4%	26.6%
BMI, kg/m^2^	31.4	31.3
Baseline PB not recorded, %	34.2%	26.6%
Baseline PB recorded, %		
Calcium acetate (CaAc)	26.6%	24.1%
Sevelamer (Sev)	36.7%	48.1%
Lanthanum carbonate	1.3%	1.3%
Switch between Sev/CaAc	1.3%	0.0%
Primary cause of ESRD, %		
Diabetes	54.4%	40.5%
Hypertension	21.5%	34.2%
Glomerulonephritis	7.6%	8.9%
Polycystic kidney	0.0%	1.3%
Other/ unknown	16.4%	15.2%
Comorbid conditions, %		
Diabetes	63.3%	63.3%
Congestive heart failure	21.5%	20.3%

Summary statistics are presented as mean or percentage

*Abbreviations: BMI* body mass index, *ESRD* end-stage renal disease, *HD* hemodialysis, *hypoAlb* hypoalbuminemic, *NhypoAlb* non-hypoalbuminemic, *PB* phosphate binder, *SO* sucroferric oxyhydroxide

^a^Patients with dialysis vintage < 120 days prior to SO initiation

**Fig. 1:**
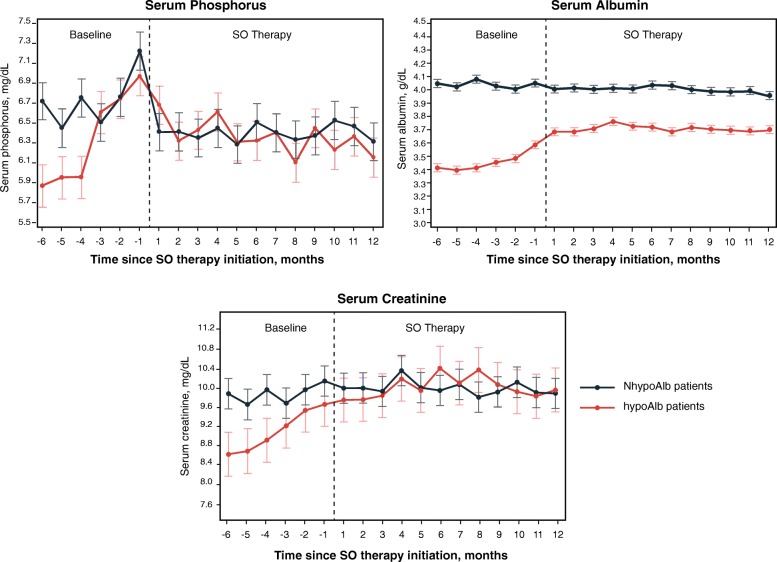
Monthly serum phosphorus, serum albumin, and serum creatinine concentrations before and after initiation of SO. Data are presented as LS means (SE). *Abbreviations: hypoAlb* hypoalbuminemic, *LS* least-square, *NhypoAlb* non-hypoalbuminemic, *SE* standard error, *SO* sucroferric oxyhydroxide

**Fig. 2:**
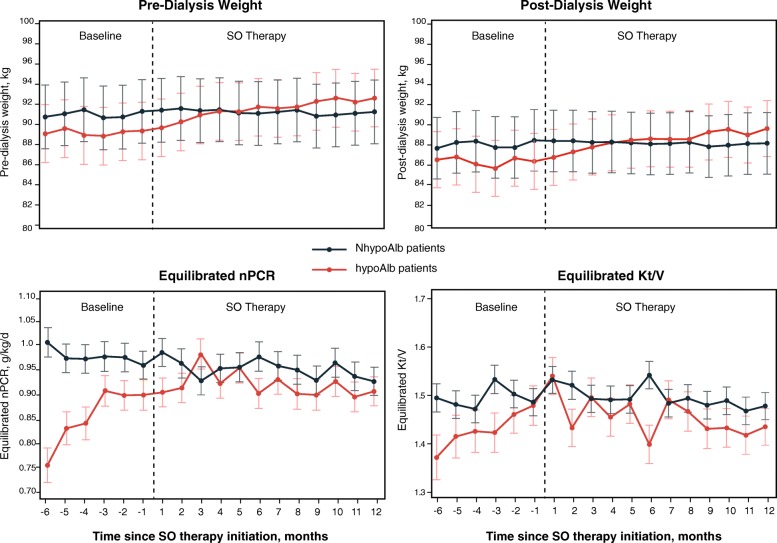
Monthly pre- and post-dialysis weights, equilibrated nPCR, and Kt/V before and after initiation of SO. Data are presented as LS means (SE). *Abbreviations: hypoAlb* hypoalbuminemic, *LS* least-square, *NhypoAlb* non-hypoalbuminemic, *nPCR* normalized protein catabolic rate, *SE* standard error, *SO* sucroferric oxyhydroxide

**Fig. 3:**
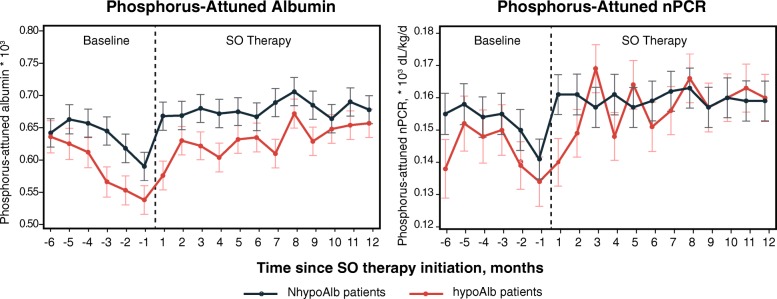
Monthly phosphorus-attuned albumin and phosphorus-attuned nPCR before and after initiation of SO therapy. Data are presented as LS means (SE). *Abbreviations: hypoAlb* hypoalbuminemic, *LS* least-square, *NhypoAlb* non-hypoalbuminemic, *nPCR* normalized protein catabolic rate, *SE* standard error, *SO* sucroferric oxyhydroxide

For each group, the monthly means of clinical parameters are presented in Fig. 1 (sP, sAlb, and serum creatinine), Fig. 2 (pre- and post-dialysis weight, nPCR, and HD adequacy), and Fig. 3 (phosphorus-attuned albumin and nPCR). The initiation of SO therapy in the hypoAlb cohort was associated with marked decreases in sP and pill burden, yet sAlb continued to rise before plateauing (Fig. 1, Table 2, baseline: 3.50 g/dL; SO follow-up: 3.69–3.74 g/dL; *P* < 0.0001). Pre-dialysis and post-dialysis weight increases were observed in the hypoAlb cohort (Fig. 2, Table 3, pre-dialysis weight: baseline, 89.1 kg; SO follow-up, 90.2–92.5 kg; *P* < 0.05 [Q1]; *P* < 0.0001 [Q2–Q4]; post-dialysis weight: baseline, 86.3 kg; SO follow-up, 87.3–89.5 kg; *P* < 0.05 [Q1]; *P* < 0.0001 [Q2–Q4]). In addition, increases in phosphorus-attuned nPCR and albumin were observed in both groups (Fig. 3).

**Table 2:** Changes in clinical parameters from baseline in hypoalbuminemic patients

Parameter	Baseline		SO therapy				Mean change from baseline
	−Q2	−Q1	Q1	Q2	Q3	Q4	
PB pills/d	7.9 [0.2]^a^	8.9 [0.2]	3.9 [0.2]^a^	4.0 [0.2]^a^	4.0 [0.2]^a^	4.1 [0.2]^a^	−4.9 [0.5]^a^
sP (mg/dL)	5.93 [0.16]^a^	6.79 [0.15]	6.48 [0.15]^c^	6.41 [0.15]^c^	6.33 [0.15]^b^	6.25 [0.15]^a^	−0.40 [0.11]^c^
sAlb (g/dL)	3.41 [0.03]^a^	3.50 [0.03]	3.69 [0.03]^a^	3.74 [0.03]^a^	3.70 [0.03]^a^	3.69 [0.03]^a^	+ 0.18 [0.03]^a^
nPCR (g/kg/d)	0.81 [0.03]^a^	0.90 [0.03]	0.93 [0.02]^ns^	0.93 [0.02]^ns^	0.91 [0.02]^ns^	0.91 [0.02]^ns^	+ 0.03 [0.02]^ns^
Phosphorus-attuned albumin (× 10^3^)	0.62 [0.01]^a^	0.55 [0.01]	0.61 [0.01]^a^	0.62 [0.01]^a^	0.64 [0.01]^a^	0.65 [0.01]^a^	+ 0.08 [0.01]^a^
Phosphorus-attuned nPCR (× 10^3^ dL/kg/d)	0.15 [0.02]^ns^	0.14 [0.02]	0.15 [0.02]^c^	0.15 [0.02]^c^	0.16 [0.02]^b^	0.16 [0.02]^a^	+ 0.02 [0.01]^a^
Pre-dialysis weight (kg)	89.2 [2.8]^ns^	89.1 [2.8]	90.2 [2.8]^c^	91.4 [2.8]^a^	91.8 [2.8]^a^	92.5 [2.8]^a^	+ 2.2 [2.2]^a^
Post-dialysis weight (kg)	86.5 [2.8]^ns^	86.3 [2.8]	87.3 [2.8]^c^	88.5 [2.8]^a^	88.9 [2.8]^a^	89.5 [2.8]^a^	+ 2.1 [1.7] ^a^
Equilibrated Kt/V	1.41 [0.03]^ns^	1.45 [0.03]	1.49 [0.03]^ns^	1.45 [0.03]^ns^	1.47 [0.03]^ns^	1.43 [0.03]^ns^	+ 0.014 [0.02]^ns^
Serum creatinine (mg/dL)	8.7 [0.4]^b^	9.4 [0.4]	9.7 [0.4]^ns^	10.0 [0.4]^b^	10.0 [0.4]^b^	9.8 [0.4]^c^	+ 0.6 [0.6]^b^
iPTH (pg/mL)	528 [43]^ns^	568 [42]	569 [41]^ns^	552 [41]^ns^	579 [41]^ns^	536 [41]^ns^	−17 [45]^ns^
Corrected calcium (mg/dL)	9.3 [0.1]^ns^	9.3 [0.1]	9.3 [0.1]^ns^	9.2 [0.1]^c^	9.2 [0.1]^c^	9.2 [0.1]^c^	−0.08 [0.1]^ns^

Summary statistics are expressed as LS means [standard errors]

*Abbreviations: iPTH* intact parathyroid hormone, *LS* least-square, *nPCR* normalized protein catabolic rate, *ns* non-significant, *PB* phosphate binder, *sAlb* serum albumin, *SO* sucroferric oxyhydroxide, *sP* serum phosphorus

All comparisons were carried out with −Q1 as the reference. ^a^*P* < 0.0001, ^b^*P* < 0.001, ^c^*P* < 0.05, ^ns^non-significant

**Table 3:** Changes in clinical parameters from baseline in non-hypoalbuminemic patients

Parameter	Baseline		SO therapy				Mean change from baseline
	−Q2	−Q1	Q1	Q2	Q3	Q4	
PB pills/d	8.4 [0.2]^c^	8.9 [0.2]	3.7 [0.2]^a^	3.8 [0.2]^a^	3.8 [0.2]^a^	3.8 [0.2]^a^	−5.0 [0.5]^a^
sP (mg/dL)	6.65 [0.16]^ns^	6.83 [0.16]	6.39 [0.16]^a^	6.41 [0.16]^a^	6.37 [0.16]^a^	6.43 [0.16]^b^	−0.51 [0.12]^a^
sAlb (g/dL)	4.05 [0.02]^ns^	4.03 [0.02]	4.01 [0.02]^ns^	4.02 [0.02]^ns^	4.01 [0.02]^ns^	3.97 [0.02]^c^	−0.02 [0.03]^ns^
nPCR (g/kg/d)	0.98 [0.02]^ns^	0.97 [0.02]	0.96 [0.02]^ns^	0.96 [0.02]^ns^	0.95 [0.02]^ns^	0.94 [0.02]^ns^	−0.02 [0.02]^ns^
Phosphorus-attuned albumin (× 10^3^)	0.65 [0.02]^c^	0.62 [0.02]	0.67 [0.02]^a^	0.67 [0.02]^a^	0.69 [0.02]^a^	0.68 [0.02]^a^	+ 0.06 [0.01]^a^
Phosphorus-attuned nPCR (× 10^3^ dL/kg/d)	0.16 [0.01]^ns^	0.15 [0.01]	0.16 [0.01]^c^	0.16 [0.01]^c^	0.16 [0.01]^b^	0.16 [0.01]^c^	+ 0.01 [0.01]^c^
Pre-dialysis weight (kg)	91.1 [3.2]^ns^	90.9 [3.2]	91.4 [3.2]^c^	91.2 [3.2]^ns^	91.2 [3.2]^ns^	91.1 [3.2]^ns^	+ 0.5 [0.4]^ns^
Post-dialysis weight (kg)	88.2 [3.1]^ns^	88.1 [3.1]	88.4 [3.1]^ns^	88.3 [3.1]^ns^	88.2 [3.1]^ns^	88.2 [3.1]^ns^	+ 0.4 [0.4]^ns^
Equilibrated Kt/V	1.48 [0.02]^ns^	1.51 [0.02]	1.52 [0.02]^ns^	1.51 [0.02]^ns^	1.49 [0.02]^ns^	1.48 [0.02]^ns^	−0.008 [0.02]^ns^
Serum creatinine (mg/dL)	9.7 [0.3]^ns^	9.8 [0.3]	9.9 [0.3]^ns^	10.0 [0.3]^ns^	9.8 [0.3]^ns^	9.8 [0.3]^ns^	+ 0.08 [0.1]^ns^
iPTH (pg/mL)	609 [64]^ns^	633 [64]	651 [63]^ns^	656 [63]^ns^	643 [63]^ns^	763 [63]^b^	+ 49 [44]^ns^
Corrected calcium (mg/dL)	9.1 [0.07]^ns^	9.2 [0.07]	9.2 [0.07]^ns^	9.1 [0.07]^ns^	9.1 [0.07]^ns^	9.1 [0.07]^ns^	−0.04 [0.05]^ns^

Summary statistics are expressed as LS means [standard errors]

*Abbreviations: iPTH* intact parathyroid hormone, *LS* least-square, *nPCR* normalized protein catabolic rate, *ns* nonsignificant, *PB* phosphate binder, *sAlb* serum albumin, *SO* sucroferric oxyhydroxide, *sP* serum phosphorus

All comparisons were carried out with −Q1 as the reference. ^a^*P* < 0.0001, ^b^*P* < 0.001, ^c^*P* < 0.05, ^ns^non-significant

## Discussion

In this retrospective cohort study, we found that the initiation of SO resulted in significant reduction in sP concentrations and daily PB pill burden in patients with low and normal sAlb levels at baseline. As observed in prior studies [16, 19–21], and independent of baseline sAlb status, SO effectively reduced sP, with a 55 to 56% decrease in mean daily PB pill burden (Table 2 and Table 3). Despite reduced sP levels following the initiation of SO, the sAlb concentration continued to rise before plateauing in patients who were hypoalbuminemic at baseline. This observation may have important clinical implications for lowering the risk of PEW and improving patient outcomes.

Baseline data demonstrated that hypoAlb patients had lower sP, PB pill burden, and nPCR than patients in the reference group (Fig. 1, Fig. 2, and Table 1), suggesting an increased reliance on protein restriction for phosphate control. Sharp increases in mean equilibrated nPCR during months − 5 to − 3 (Fig. 2), increases in mean serum creatinine during months − 5 to − 1 (Fig. 1), and subsequent increases in sAlb in month − 1 (Fig. 1) suggest that patients increased their protein intake at the “expense” of elevated sP (Fig. 1), as also evidenced by decreasing phosphorus-attuned albumin (Fig. 3).

There are no data or mechanistic hypotheses to suggest that SO therapy directly impacts protein handling or appetite in patients. Instead, the improvements in sAlb and progressive weight increases observed in the hypoAlb cohort during SO therapy likely resulted from continued increases in protein intake, as suggested by small increases in phosphorus-attuned nPCR. Such changes may be the result of dietary counseling (e.g., accompanying the initiation of SO and sP lowering). It is also possible that the higher baseline PB pill burden impaired appetite and overall nutritional intake as a result of gastrointestinal side effects [11], and the switch to SO may have allowed for improved nutritional intake. In a recent meta-analysis, sevelamer was associated with 32% more gastrointestinal side effects than SO (*P* = 0.0001) [22]. Furthermore, in 2 active-controlled, pivotal trials, SO was associated with fewer reports of decreased appetite than sevelamer (1.9% vs 4.3%) [23]. The reduced pill burden and increased potency [11] associated with SO and/or its non-resin-based formulation (52/79 hypoAlb patients on baseline PB: 56% sevelamer, 40% calcium acetate, 4% other) may not negatively impact appetite to the extent observed with other PBs.

Increasing sAlb has been reported to improve patient outcomes. For instance, it has been proposed that increasing sAlb > 3.8 g/dL among US HD patients might prevent ~ 10,000 deaths annually [24]. Temporal decreases in sP with concomitant increases in sAlb have been associated with a survival benefit of 8 to 9% [4]. More recently, use of a PB that improves nutritional markers such as sAlb and nPCR was associated with a significant reduction in mortality [25]. There are also data to suggest that allowing unrestricted dietary protein intake by HD patients may improve survival [5].

The results of the present study highlight the challenging nature of phosphate control among HD populations. While the percentage of patients attaining sP < 5.5 mg/dl and < 4.5 mg/dl increased in our study, the therapeutic approach to managing and preventing hyperphosphatemia should not be limited to PB pharmacotherapy. Appropriate dietary counseling and HD adequacy should also be employed [26].

The observational nature of this analysis provides valuable information from a real-world cohort of patients treated in HD practices across the United States, but the results should be interpreted in the context of several limitations. Although a reference group was included, matching was performed only on the basis of 5 characteristics and therefore prevented the establishment of an appropriate control group. Differences in baseline PB use and dietary status did not influence inclusion in the reference group. A causal relationship between SO and improved nutritional markers cannot be established, given the observational nature of this analysis. Beyond the initiation of therapy with SO, changes in nutritional counseling or concomitant illness may have influenced observed laboratory changes. Additionally, the analysis did not account for factors capable of impacting sAlb, such as acute-phase reactants or residual renal function [7, 27]. Future prospective studies may consider the addition of bioimpedance and dietary intake measures as a follow-up to this retrospective database study. Body composition data from bioimpedance measurements may be helpful to investigate the nature of the body weight increase observed in hypoalbuminemic patients (e.g. lean tissue mass, adipose tissue mass, excess fluid), and self-reported measures of dietary intake such as a dietary diary may help to fully capture dietary changes, and dietary intake of proteins.

## Conclusion

SO was associated with significant reductions in sP levels and PB pill burden in a real-world cohort of HD patients. As evidenced by sustained improvements in nutritional status, SO may be particularly helpful for the control of sP in those HD patients exhibiting evidence of protein malnutrition (i.e., reduced sAlb) by allowing moderation of dietary protein restriction.

## Data Availability

We encourage investigators interested in data sharing and collaboration to contact the corresponding author.

## Abbreviations

BMI: Body mass index
ESRD: End-stage renal disease
HD: Hemodialysis
hypoAlb: Hypoalbuminemic
iPTH: Intact parathyroid hormone
LS: Least-square
NhypoAlb: Non-hypoalbuminemic
nPCR: Normalized protein catabolic rate
ns: Non-significant
PB: Phosphate binder
PEW: Protein-energy wasting
sAlb: Serum albumin
SE: Standard error
SO: Sucroferric oxyhydroxide
sP: Serum phosphorus
